# Individualized assessment of risk and overall survival in patients newly diagnosed with primary osseous spinal neoplasms with synchronous distant metastasis

**DOI:** 10.3389/fpubh.2022.955427

**Published:** 2022-08-22

**Authors:** Yuexin Tong, Zhangheng Huang, Liming Jiang, Yangwei Pi, Yan Gong, Dongxu Zhao

**Affiliations:** ^1^Department of Orthopedics, The China-Japan Union Hospital of Jilin University, Changchun, China; ^2^Department of Orthopedics, Orthopedic Research Institute, West China Hospital, Sichuan University, Chengdu, China

**Keywords:** primary osseous spinal neoplasms, nomogram, distant metastasis, SEER database, prognosis

## Abstract

**Background:**

The prognosis of patients with primary osseous spinal neoplasms (POSNs) presented with distant metastases (DMs) is still poor. This study aimed to evaluate the independent risk and prognostic factors in this population and then develop two web-based models to predict the probability of DM in patients with POSNs and the overall survival (OS) rate of patients with DM.

**Methods:**

The data of patients with POSNs diagnosed between 2004 and 2017 were extracted from the Surveillance, Epidemiology, and End Results (SEER) database. Univariate and multivariate logistics regression analyses were used to study the risk factors of DM. Based on independent DM-related variables, we developed a diagnostic nomogram to estimate the risk of DM in patients with POSNs. Among all patients with POSNs, those who had synchronous DM were included in the prognostic cohort for investigating the prognostic factors by using Cox regression analysis, and then a nomogram incorporating predictors was developed to predict the OS of patients with POSNs with DM. Kaplan–Meier (K-M) survival analysis was conducted to study the survival difference. In addition, validation of these nomograms were performed by using receiver operating characteristic (ROC) curves, the area under curves (AUCs), calibration curves, and decision curve analysis (DCA).

**Results:**

A total of 1345 patients with POSNs were included in the study, of which 238 cases (17.70%) had synchronous DM at the initial diagnosis. K-M survival analysis and multivariate Cox regression analysis showed that patients with DM had poorer prognosis. Grade, T stage, N stage, and histological type were found to be significantly associated with DM in patients with POSNs. Age, surgery, and histological type were identified as independent prognostic factors of patients with POSNs with DM. Subsequently, two nomograms and their online versions (https://yxyx.shinyapps.io/RiskofDMin/ and https://yxyx.shinyapps.io/SurvivalPOSNs/) were developed. The results of ROC curves, calibration curves, DCA, and K-M survival analysis together showed the excellent predictive accuracy and clinical utility of these newly proposed nomograms.

**Conclusion:**

We developed two well-validated nomograms to accurately quantify the probability of DM in patients with POSNs and predict the OS rate in patients with DM, which were expected to be useful tools to facilitate individualized clinical management of these patients.

## Introduction

Primary osseous spinal neoplasms (POSNs) are a rare group of spinal malignancies, which represents approximately 5% of all bone neoplasms (1, 2). Although bone sarcoma can occur in various osseous regions throughout the body, tumors of the spine and surrounding structures generally elicit more debilitating consequences due to severe focal pain and neurologic morbidity (3). Currently, *en bloc* resection is recognized as the most effective method for treatment of POSNs (4, 5), and the 5-year survival rate of patients with POSNs following aggressive surgery has been improved to 65%−70% (6, 7).

As with other common malignancies, distant metastasis (DM) was also reported to be a significant factor affecting the survival rate of patients with POSNs (8), and the occurrence of metastatic disease would greatly decrease the survival time of these patients (9, 10). More frustratingly, these metastases usually remain asymptomatic until they progress to the point of destroying the organ function and are therefore easily overlooked. In addition, it was reported that patients with synchronous DM had a significantly worse prognosis than those with metachronous DM. The time of appearance of DM should be regarded as an important indicator for the biological invasiveness of the primary tumor (11–13). Therefore, the importance of early detection of synchronous DM in patients with POSNs and individualized survival prediction of patients with metastatic POSNs is self-evident for optimizing medical decision-making. Specifically, by identifying patients at high risk for metastasis, physicians can recommend more frequent monitoring programs for them, such as chest thin-section computed tomography (CT), the whole-body 99Tc methylene diphosphonate (MDP) bone scan, positron emission computed tomography (PET/CT)(14, 15); for those patients with POSNs who have developed synchronous DM, an accurate prognostic assessment contributes to improving clinical management. The nomogram model, which transforms traditional statistical predictive models into visualized probability estimates tailored to each patient, is suitable for cancer prognostic studies (16–18).

Within the last few years, several studies have developed nomograms for primary bone tumors of the spine (19, 20). Zhou et al. have retrospectively analyzed 1096 patients with primary spine malignancies and provided statistical evidence of their clinical characteristics and prognostic predictors (21). Nevertheless, until now, most of the prognostic studies have mainly focused on the whole group of patients with POSNs, leading to the scarcity of prognostic information on patients with POSNs with DM as a specific cohort (8, 22). Furthermore, some other critical issues also remain to be illuminated in patients with POSNs with DM. First, it is veiled that patients with which clinicopathologic features are more likely to develop distant metastases; second, the therapeutic modality which is effective once patients developed metastasis remains to be determined. These problems are of vital importance and can serve as pivotal reference for clinicians to optimize clinical interventions for patients with POSNs, especially for those who have developed metastasis. It is therefore necessary to study a cohort of patients with POSNs with metastases in order to elucidate these issues.

In the present study, we aimed to perform a population-based analysis of this rare subgroup to develop two online prediction models for quantifying the probability of DM in patients with POSNs and predicting the survival rate of patients with POSNs with DM.

## Methods

### Study population

**Figure 1:**
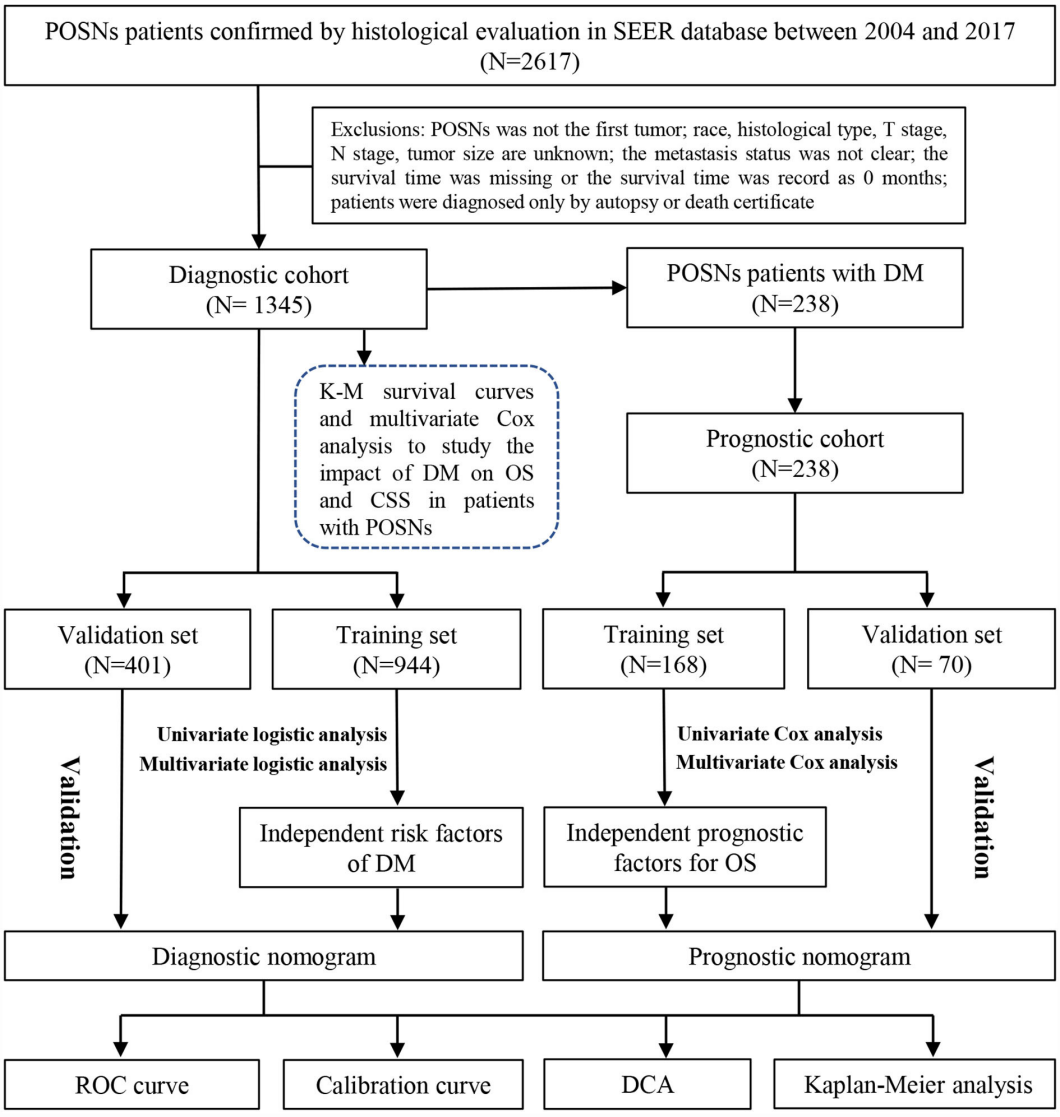
Patients' selection and workflow of this study.

The Surveillance Epidemiology and End Results (SEER) database is the largest publicly available cancer dataset, representing approximately 28% of the U.S. population (23). The research data of patients were downloaded from the SEER 18 registries (www.seer.cancer.gov) of the National Cancer Institute *via* SEER^*^Stat 8.4.1. Since the SEER database did not publish personally identifiable information, this study did not require the review of the ethic committee of the China-Japan Union Hospital of Jilin University. According to the field of “Primary Site-labeled,” patients histologically diagnosed with POSNs between 2004 and 2017 were included in this study. Patients would be excluded from the study if they met any of the following criteria: (1) race, histological type, T stage, N stage, and tumor size were unknown; (2) the POSN was not a primary tumor; (3) the metastasis status was not clear; (4) the survival time was missing or the survival time was record as 0 month; and (5) POSN was diagnosed only by autopsy or after death. The primary outcome included overall survival (OS) and cancer-specific survival (CSS). OS was defined as survival from diagnosis to death of any cause, and CSS was explained as the span from the diagnosis date until death only caused by POSNs. Ultimately, 1,345 patients with POSNs were included to in this study, 238 of whom presented synchronous DM. All eligible patients were enrolled to form the diagnostic cohort to identify the risk factors of DMs and construct a diagnostic nomogram for quantification of the probability of DM. Subsequently, the 238 patients with POSNs with DM were further included in the prognostic cohort to explore the prognostic factors and establish a prognostic nomogram for OS prediction. The patient selection and workflow of this study are illustrated in Figure 1.

### Data selection

In this study, eight variables were used to examine the relationship with the occurrence of DM: age at diagnosis, race, sex, tumor grade, histological type, T stage, N stage, and tumor size. For determining independent prognostic factors for patients with POSNs with DM, treatment information was also collected, including surgery, radiotherapy, and chemotherapy. Age at diagnosis and tumor size were transfer categorical variables (age: <40 years, 40–59 years, and ≥ 60 years; tumor size: <5 cm, 5-10 cm, and >10 cm), respectively. The type of histology was divided into five categories using the International Classification of Diseases for Oncology, third edition (ICD-O-3) Hist/behav: osteosarcoma (9180-9187 and 9192-9194), chondrosarcoma (9220, 9221, 9230, 9231, and 9240-9243), Ewing sarcoma (9260), and chordoma (9370-9372) including conventional, dedifferentiated, and others. Survival data were also downloaded for survival analysis. In addition, grades I and II (well-differentiated and differentiated) were classified as low grade, and grades III and IV (poorly differentiated and undifferentiated) were classified as high grade.

### Statistical analyses

All statistical analyses in this study were performed in SPSS 26.0 and R software version 4.0.2 (https://www.r-project.org/). *P*-values <0.05 (both sides) were considered statistically significant. First, Kaplan–Meier (K–M) survival analysis was performed to study the impact of DM on OS and CSS in patients with POSNs. Then, multivariate Cox regression analysis was used to further confirm whether DM served as an independent prognostic factor for patients with POSNs. In both study cohorts, the patients were randomly divided into a training set and a validation set, with a ratio of 7:3 using R software. The chi-square (χ2) test or Fisher exact test was used to compare variables between the two sets. We used univariate logistic regression analysis to find out DM-related factors. Variables with *P* < 0.05 in univariate analysis were further included in the multivariate logistic regression analysis. Then, independent risk factors (*P* < 0.05 in multivariate analysis) for DM in patients with POSNs were eventually determined. Odds ratios (ORs) and 95% confidence intervals (CIs) were also calculated to show the relevance between clinical characteristics and the development of DM.

In addition, Cox regression analysis was utilized to determine the independent prognostic factors in patients with POSNs with DM. Variables with *P* < 0.05 in the univariate analysis were used in multivariate analysis. Similarly, the impact of the predictor on OS was illustrated by using the hazard ratio (HR) and corresponding 95% CIs.

Afterward, based on determined independent risk factors and prognostic factors, the corresponding nomogram was developed by the “rms” package in R software. Moreover, the two web-based nomograms were further developed using the “DynNom” software package to accurately calculate the probability of DM in patients newly diagnosed with POSNs and the OS rate of patients with synchronous DM. The discriminative ability of the model was evaluated by the receiver operating characteristic (ROC) curve and the value of area under curve (AUC). Calibration curves were plotted to assess the consistency between observational values and predicted values, and decision curve analysis (DCA) was performed to evaluate the clinical value of the nomograms. In addition, net reclassification improvement (NRI) and integrated discrimination improvement (IDI) were used to study whether the model was more accurate in evaluating prognosis than American Joint Committee on Cancer (AJCC) TNM staging system or not. Furthermore, the individual total point of each patient with POSNs with DM was calculated based on the prognostic nomogram. According to the median total point, the patients were assigned into high- and low-risk groups. K–M survival analysis with the log-rank test was conducted to study the difference of OS between the two groups.

## Result

### Clinicopathologic characteristics of patients with POSNs

**Figure 2:**
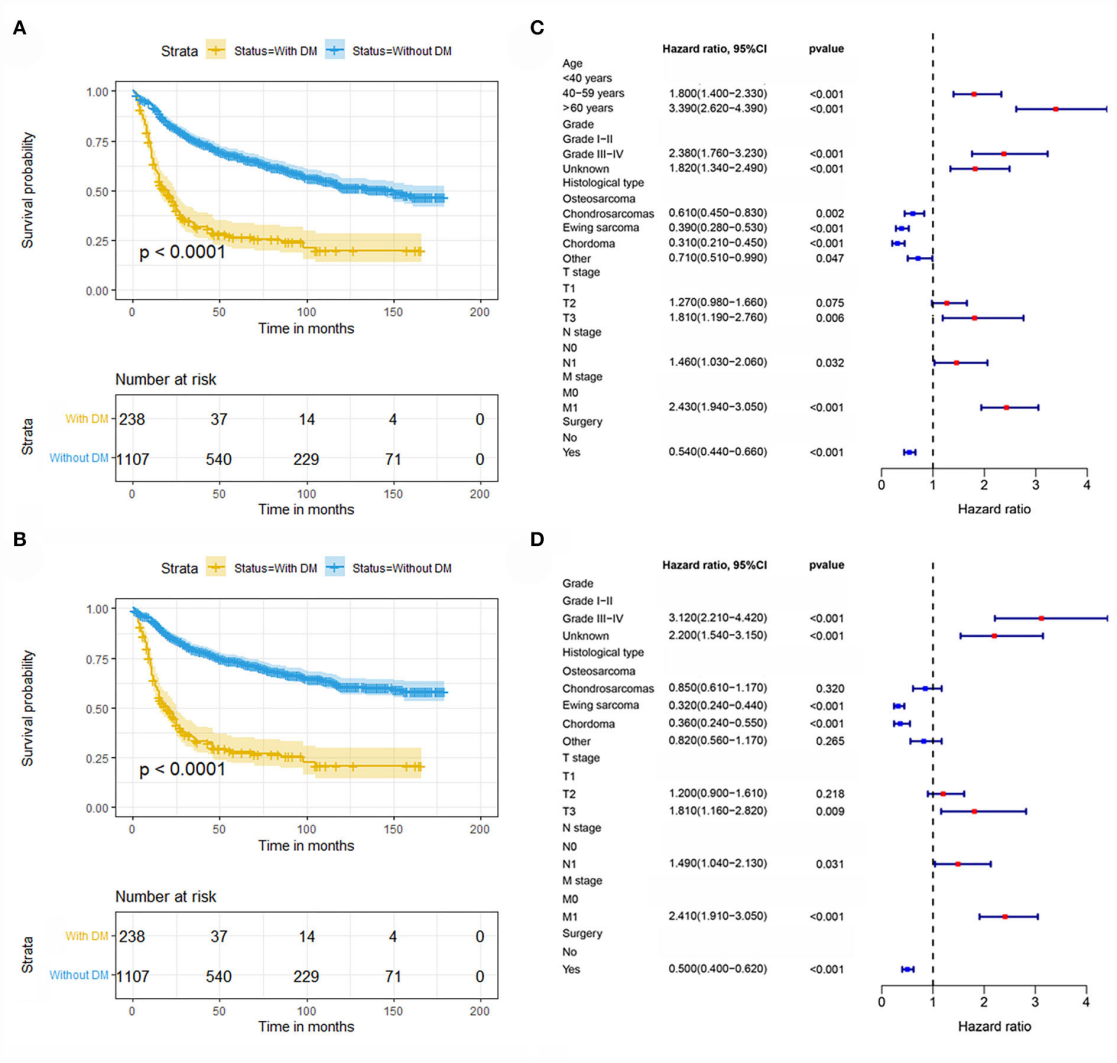
Kaplan–Meier survival analysis to investigate the impact of DM on OS **(A)** and CSS **(B)** in patients with POSNs. The forest plot showed the results of multivariate Cox regression analysis, where DM was significantly associated with OS **(C)** and CSS **(D)** of patients with POSNs.

In our study, 1345 patients with POSNs were finally enrolled. Table 1 describes the demographic and clinicopathologic characteristics of the patients with POSNs with or without DM, with 798 (59.33%) male and 547 (40.67%) female patients. The predominant ethnicity was white (*N* = 1145, 85.13%). The distribution of tumor grade was almost equal, with 358 (26.62%) well-differentiated and 353 (26.25%) poorly differentiated patients. In terms of tumor size, the majority of patients (*N* = 595, 44.24%) had 5- to 10-cm tumors. In addition, the patients with POSNs often had chondrosarcomas (*N* = 432, 32.12%) and stage N0 (*N* = 1285, 95.54%). Meanwhile, the chi-square test showed that the deviation was completely randomized (Table 1). As shown in Figure 2, K-M survival curves showed that the patients with POSNs with DM had poorer prognosis than those without DM (*P* < 0.05) (Figures 2A,B), and the multivariate Cox regression analysis further confirmed that DM was significantly associated with OS and CSS of patients with POSNs (Figures 2C,D).

**Table 1:** The demographic and clinicopathological characteristics of the POSNs patients with or without DM.

**Variables**	**Overall cohort (*N =* 1345, %)**	**Training set** ** (*N =* 944, %)**	**Validation set (*N =* 401, %)**	***P*–value**
Age				
<40 years	573 (42.60)	404 (42.80)	169 (42.14)	0.9739
40–59 years	378 (28.10)	264 (27.97)	114 (28.43)	
≥60 years	394 (29.29)	276 (29.24)	118 (29.43)	
Sex				
Female	547 (40.67)	374 (39.62)	173 (43.14)	0.2532
Male	798 (59.33)	570 (60.38)	228 (56.86)	
Race				
Black	94 (6.99)	69 (7.31)	25 (6.23)	0.7647
Other	106 (7.88)	75 (7.94)	31 (7.73)	
White	1,145 (85.13)	800 (84.75)	345 (86.03)	
Histological type				
Osteosarcoma	180 (13.38)	129 (13.67)	51 (12.72)	0.463
Chondrosarcomas	432 (32.12)	294 (31.14)	138 (34.41)	
Ewing sarcoma	284 (21.12)	193 (20.44)	91 (22.69)	
Chordoma	337 (25.06)	247 (26.17)	90 (22.44)	
Other	112 (8.33)	81 (8.58)	31 (7.73)	
Grade				
Low (well differentiation)	358 (26.62)	250 (26.48)	108 (26.93)	0.9791
High (poor differentiation)	353 (26.25)	249 (26.38)	104 (25.94)	
Unknown	634 (47.14)	445 (47.14)	189 (47.13)	
T stage				
T1	646 (48.03)	454 (48.09)	192 (47.88)	0.9788
T2	657 (48.85)	460 (48.73)	197 (49.13)	
T3	42 (3.12)	30 (3.18)	12 (2.99)	
N stage				
N0	1285 (95.54)	906 (95.97)	379 (94.51)	0.2971
N1	60 (4.46)	38 (4.03)	22 (5.49)	
Tumor size				
<5 cm	283 (21.04)	212 (22.46)	71 (17.71)	0.093
5–10 cm	595 (44.24)	403 (42.69)	192 (47.88)	
>10 cm	467 (34.72)	329 (34.85)	138 (34.41)	

### Risk factors in development of DM and diagnostic nomogram

**Figure 3:**
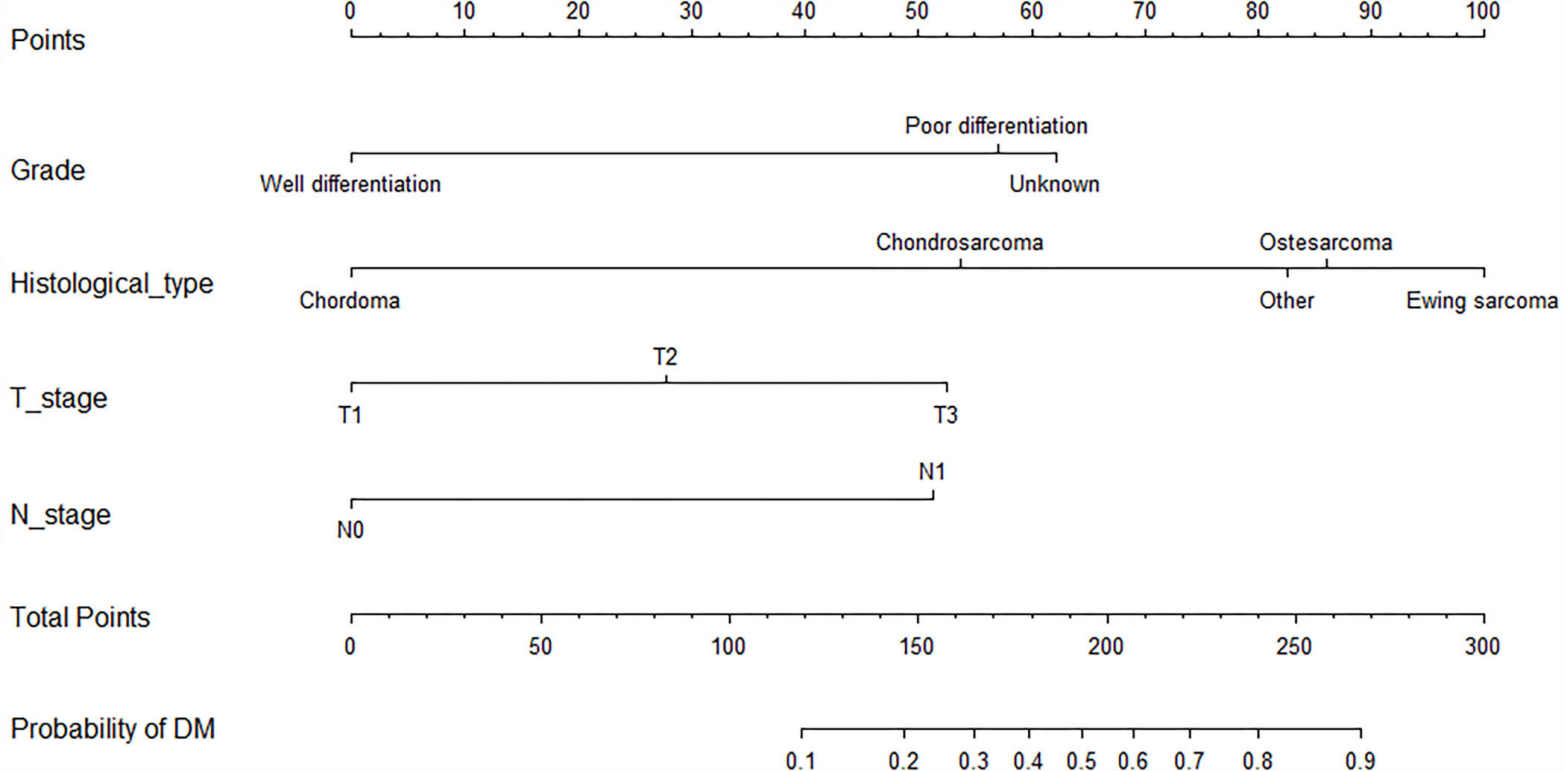
Diagnostic nomogram for quantifying the probability of DM in patients with POSNs.

As shown in Table 2, the univariate logistics analysis suggested that patients with the histological type of Ewing sarcoma, poor tumor differentiation, advanced age, stage N1, higher T stage, and larger tumor size tended to present with DM at initial diagnosis. Based on the result of the multivariate analysis, grade, histological type, T stage, and N stage (all *p* < 0.05) were finally determined as the independent risk factors for DM in newly diagnosed patients with POSNs. Subsequently, a diagnostic nomogram was developed to quantify the probability of DM in patients with POSNs (Figure 3), and the online version could be accessible *via* https://yxyx.shinyapps.io/RiskofDMin/.

**Table 2:** Univariate and multivariate logistic analysis to determine the independent risk factors of DM in patient with POSNs.

**Variables**	**Univariate analysis**			**Multivariate analysis**		
	**OR**	**95%CIs**	***P*–value**	**OR**	**95%CIs**	***P*–value**
Age						
<40 years	Reference			Reference		
40–59 years	0.34	0.22–0.52	<0.001	0.98	0.57–1.71	0.953
≥60 years	0.25	0.16–0.4	<0.001	0.93	0.5–1.72	0.815
Sex						
Female	Reference					
Male	0.92	0.66–1.3	0.647			
Race						
Black	Reference					
Other	1.5	0.66–3.4	0.331			
White	1.01	0.53–1.93	0.982			
Histological type						
Osteosarcoma	Reference			Reference		
Chondrosarcomas	0.17	0.09–0.3	<0.001	0.39	0.19–0.77	0.007
Ewing sarcoma	1.7	1.06–2.73	0.027	1.48	0.85–2.58	0.17
Chordoma	0.07	0.03–0.16	<0.001	0.08	0.03–0.21	<0.001
Other	0.86	0.46–1.6	0.633	0.93	0.47–1.83	0.823
Grade						
Low (well differentiation)	Reference			Reference		
High (poor differentiation)	13.85	6.22–30.82	<0.001	5.43	2.19–13.44	<0.001
Unknown	9.05	4.12–19.85	<0.001	6.29	2.47–16	<0.001
T stage						
T1	Reference			Reference		
T2	2.79	1.92–4.05	<0.001	1.74	0.96–3.15	0.067
T3	7.76	3.56–16.92	<0.001	4.12	1.62–10.47	0.003
N stage						
N0	Reference			Reference		
N1	7.92	4.04–15.53	<0.001	4.67	2.16–10.09	<0.001
Tumor size						
<5 cm	Reference			Reference		
5–10 cm	2.29	1.31–4.01	0.004	1.69	0.87–3.3	0.122
>10 cm	4.06	2.33–7.06	<0.001	1.86	0.81–4.25	0.142

**Figure 4:**
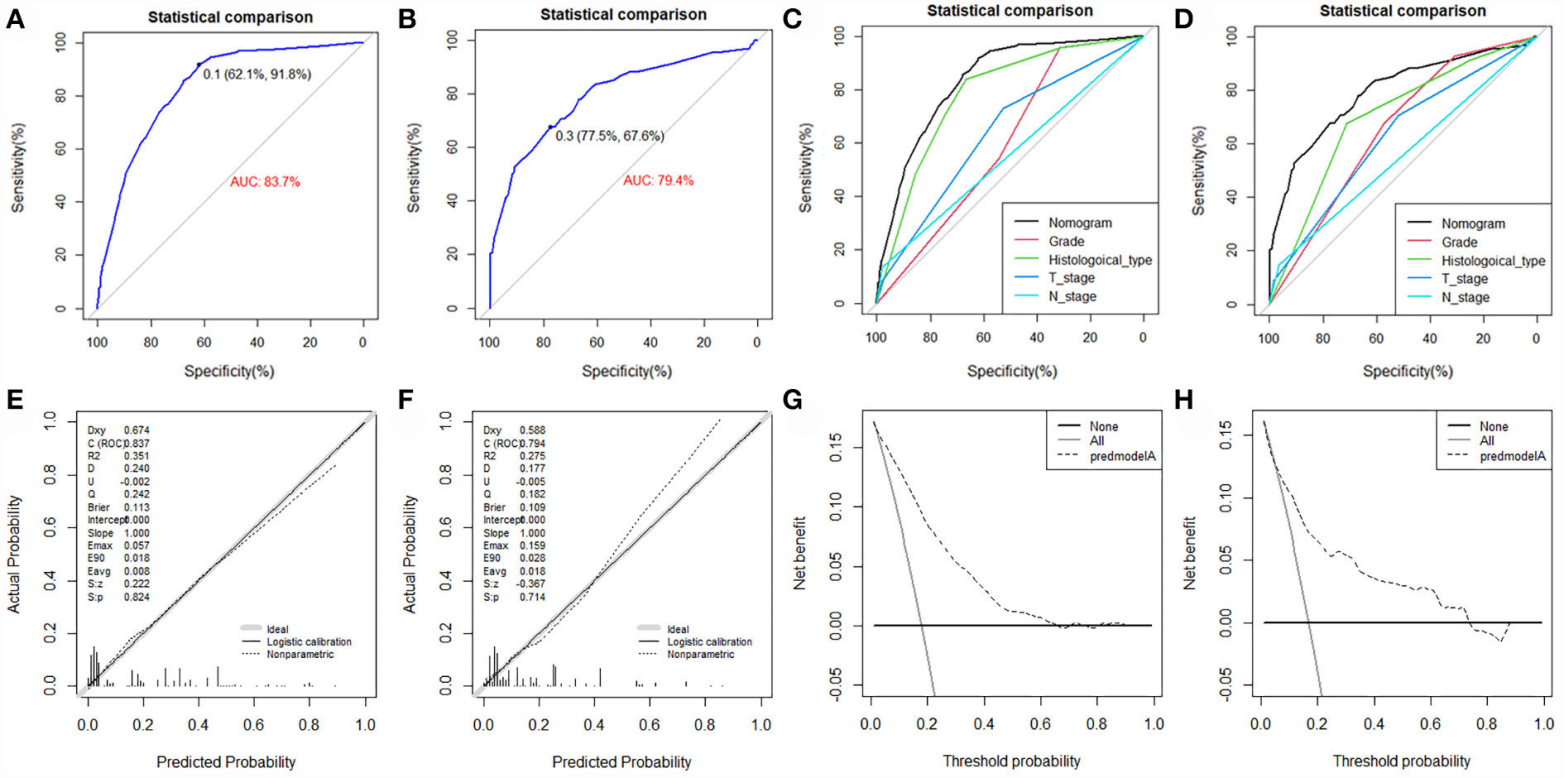
ROC curve **(A)**, calibration curve **(E)**, and DCA curve **(G)** of the training set, and the ROC curve **(B)**, calibration curve **(F)**, and DCA curve **(H)** of the validation set. Comparison of AUC between nomogram and all predictors in the training set **(C)** and validation set **(D)**.

**Figure 5:**
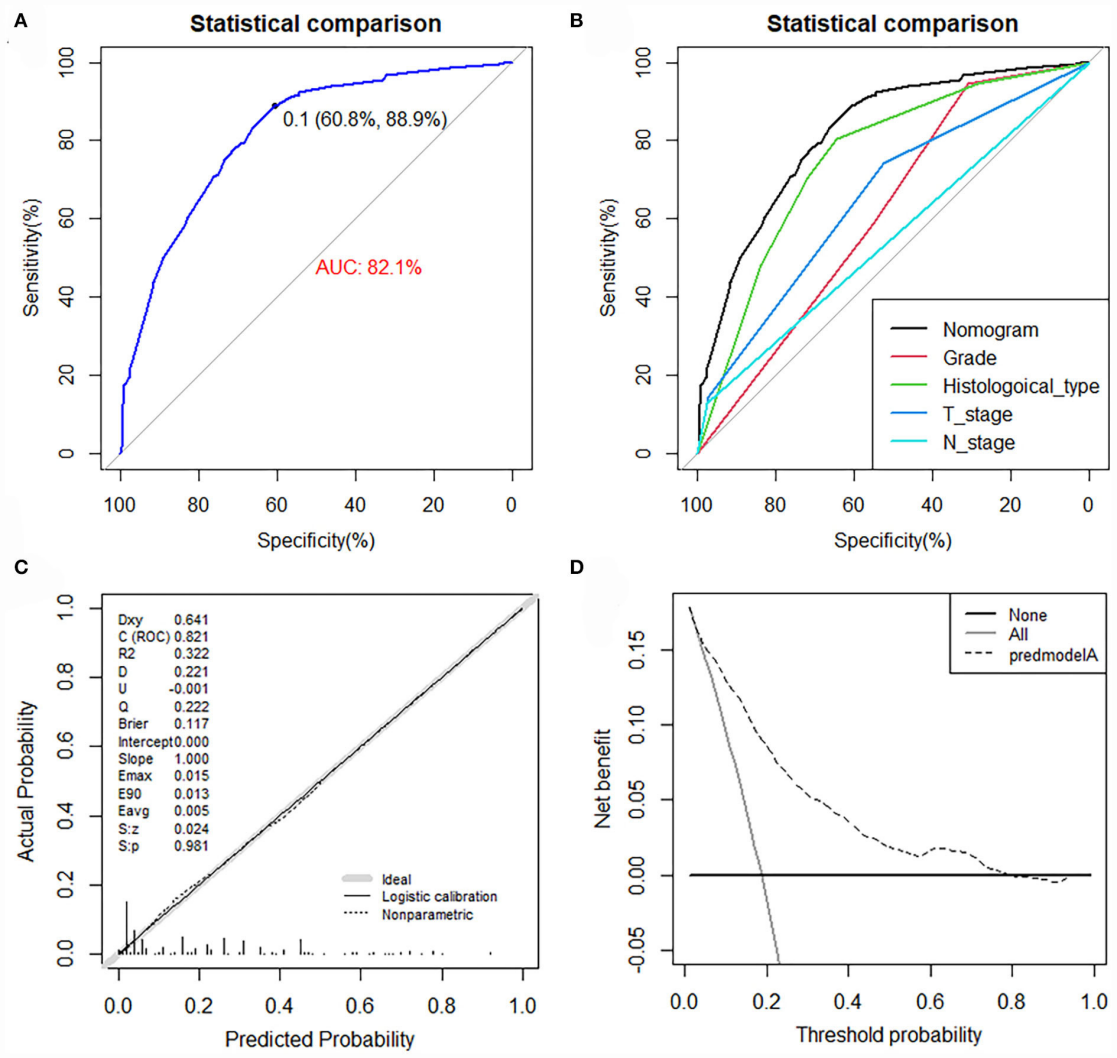
ROC curve **(A)**, comparison of AUC between the nomogram and all predictors **(B)**, calibration curve **(C)**, and DCA curve **(D)** of the expanded validation set.

The value of AUC was 0.837 (95% CI, 0.807-0.867) in the training set and 0.794 (95% CI, 0.729-0.858) in the validation set, indicating good discrimination of this model (Figures 4A,B). More importantly, ROC curves were also generated for each of the independent risk factors, with the AUCs for individual predictors significantly lower than the nomogram (*p* < 0.05), implying that the predictive accuracy of the comprehensive model was superior to that of the clinicopathologic features alone (Figures 4C,D). The favorable calibration curve indicated that the prediction by the nomogram was highly consistent with the actual observation (Figures 4E,F). In addition, the result of DCA showed that the predictive nomogram had high net benefits, meaning that it had good clinical implementation significance in predictive DM in newly diagnosed patients with POSNs (Figures 4G,H). Furthermore, due to the rarity of POSNs, it was relatively difficult to collect sufficient cases from a single institution for external validation of the nomogram, so we went back to the database and re-selected suitable patients with complete histological type, grade stage, T stage, N stage, and M stage. A total of 1450 patients were obtained to constitute an expanded validation set. The value of AUC of this set was 0.821 (95% CI: 0.794–0.847), and the calibration curve and DCA curve again confirmed the excellent predictive accuracy and clinical utility of the novel model (Figure 5).

### Prognostic factors and nomogram in patients with POSNs with DM

**Figure 6:**
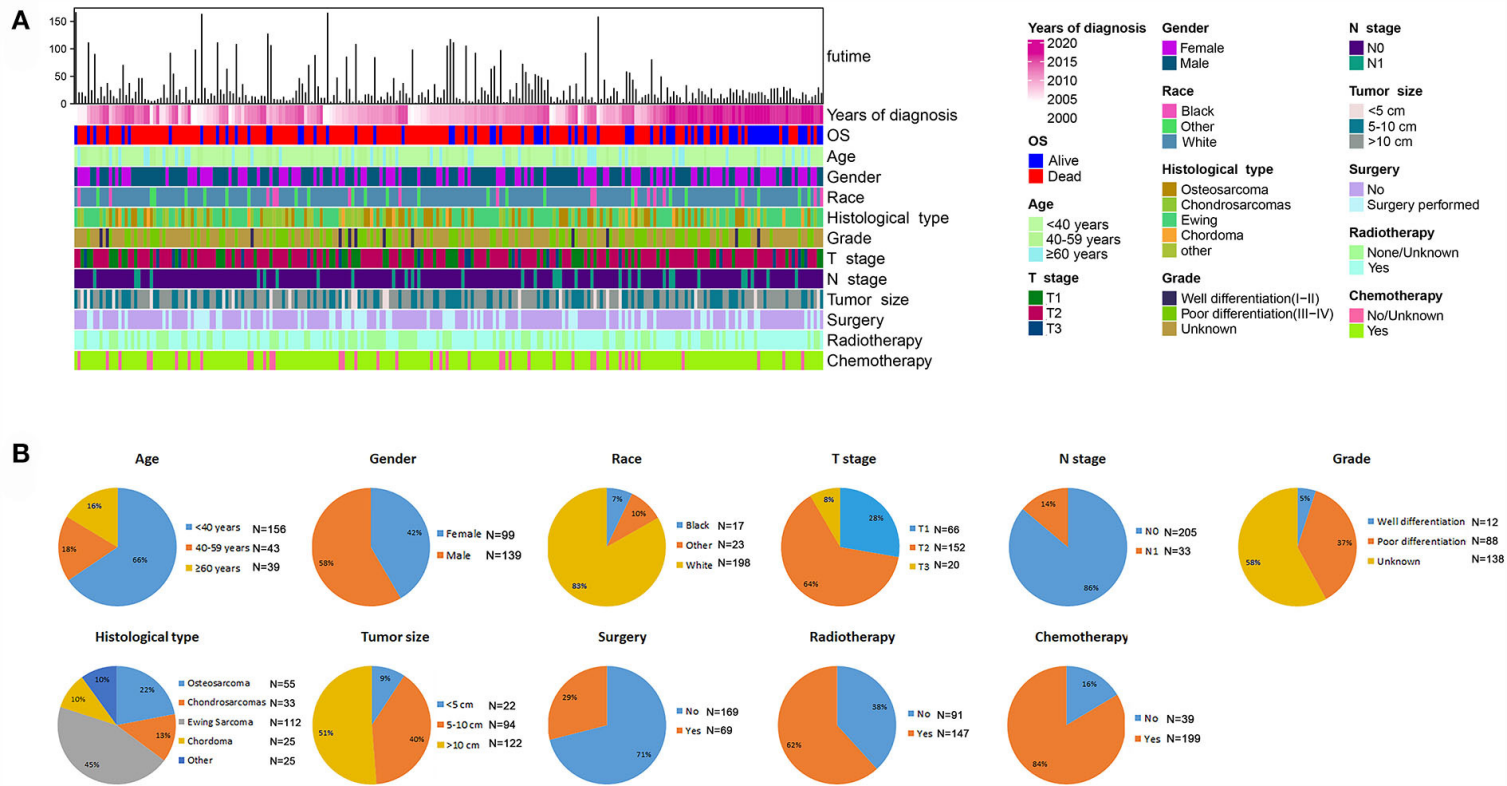
Integrated bar plot and heatmap of demographics information, tumor characteristics, and clinical outcomes of patients with POSNs with DM **(A)**. Pie chart of variables in the patients with POSNs with DM **(B)**.

Among the included patients with POSNs, DM occurred in 238 cases, accounting for 17.70%. The baseline data of patients with POSNs with DM are shown in Table 3 and Figure 6. A total of 69 (28.99%) patients received surgery, 147 (61.76%) received radiotherapy, and 199 (83.61%) received chemotherapy. The chi-square test demonstrated that the differences of all variables were not found to be statistically significant between the two sets.

**Table 3:** The baseline data of POSNs patients with DM.

**Variables**	**Overall cohort (*N =* 238, %)**	**Training set (*N =* 168, %)**	**Validation set (*N =* 70, %)**	***P*–value**
Age				0.6073
<40 years	156 (65.55)	109 (64.88)	47 (67.14)	
40–59 years	43 (18.07)	29 (17.26)	14 (20.00)	
≥ 60 years	39 (16.39)	30 (17.86)	9 (12.86)	
Sex				0.6899
Female	99 (41.60)	68 (40.48)	31 (44.29)	
Male	139 (58.40)	100 (59.52)	39 (55.71)	
Race				0.5577
Black	17 (7.14)	12 (7.14)	5 (7.14)	
Other	23 (9.66)	14 (8.33)	9 (12.86)	
White	198 (83.19)	142 (84.52)	56 (80.00)	
Histological type				0.4098
Osteosarcoma	55 (23.11)	39 (23.21)	16 (22.86)	
Chondrosarcomas	33 (13.87)	23 (13.69)	10 (14.29)	
Ewing sarcoma	112 (47.06)	81 (48.21)	31 (44.29)	
Chordoma	13 (5.46)	11 (6.55)	2 (2.86)	
Other	25 (10.50)	14 (8.33)	11 (15.71)	
Grade				0.9422
Low (well differentiation)	12 (5.04)	9 (5.36)	3 (4.29)	
High (poor differentiation)	88 (36.97)	62 (36.90)	26 (37.14)	
Unknown	138 (57.98)	97 (57.74)	41 (58.57)	
T stage				0.1208
T1	66 (27.73)	47 (27.98)	19 (27.14)	
T2	152 (63.87)	103 (61.31)	49 (70.00)	
T3	20 (8.40)	18 (10.71)	2 (2.86)	
N stage				0.9325
N0	205 (86.13)	144 (85.71)	61 (87.14)	
N1	33 (13.87)	24 (14.29)	9 (12.86)	
Tumor size				0.4579
<5 cm	22 (9.24)	13 (7.74)	9 (12.86)	
5–10 cm	94 (39.50)	67 (39.88)	27 (38.57)	
>10 cm	122 (51.26)	88 (52.38)	34 (48.57)	
Surgery				0.9485
No	169 (71.01)	120 (71.43)	49 (70.00)	
Yes	69 (28.99)	48 (28.57)	21 (30.00)	
Radiotherapy				0.4233
None	91 (38.24)	61 (36.31)	30 (42.86)	
Yes	147 (61.76)	107 (63.69)	40 (57.14)	
Chemotherapy				0.991
No	39 (16.39)	27 (16.07)	12 (17.14)	
Yes	199 (83.61)	141 (83.93)	58 (82.86)	

**Figure 7:**
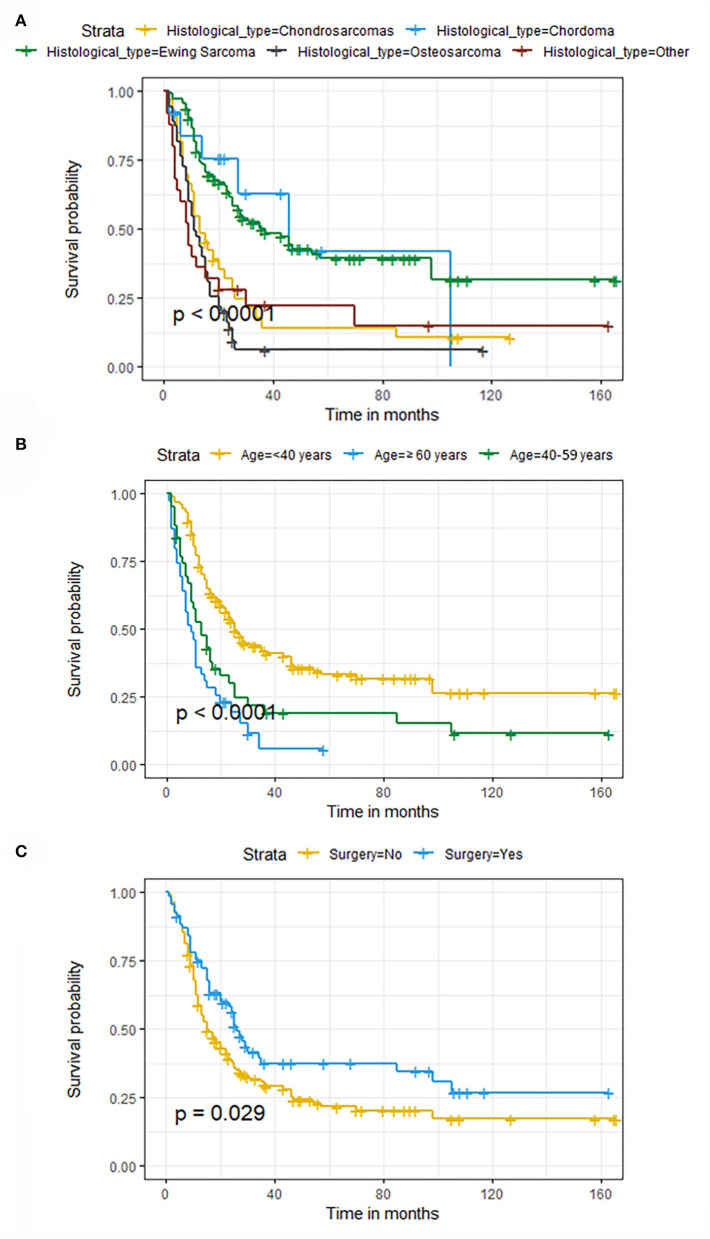
Kaplan–Meier survival curves of variables were performed for patients with POSNs with DM. **(A)** Histological type, **(B)** age, **(C)** surgery.

**Figure 8:**
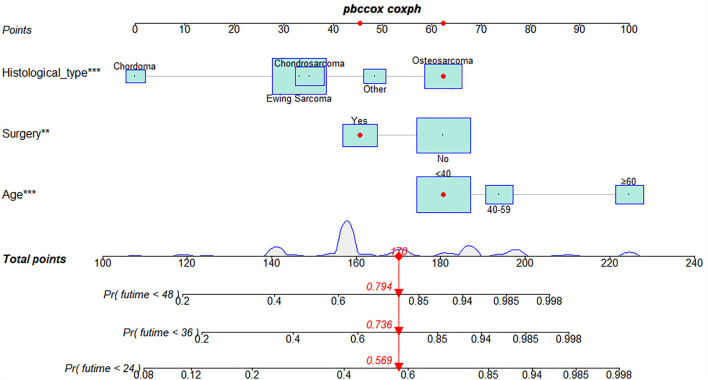
Prognostic nomogram in predicting 24-, 36-, and 48-month OS for patients with POSNs with DM. *, *** is the value of significance.

The univariate and multivariate Cox regression analyses were used to screen robust prognostic factors, which revealed that age, histological type, and surgery (all *P* < 0.05) were significantly associated with the survival rate of patients with POSNs with DM (Table 4). Consistent with the result of Cox regression analysis, Kaplan–Meier survival analysis also showed that clinical factors (histological type, age, and surgery) were significantly associated with OS (Figure 7). Then, a prognostic nomogram incorporating the aforementioned predictors was developed to predict the OS rate at 24, 36, and 48 months (Figure 8), which could be accessible *via* https://yxyx.shinyapps.io/SurvivalPOSNs/. The nomogram showed that the histological type contributed most to OS, followed by age. When using the nomogram, the individual patient values were located on each variable axis, and then a red line was drawn upward to determine the number of points obtained for each variable value. The sum of these numbers is located on the total point axis, and a red line is drawn downward to the survival axis to determine the probabilities of OS at 24, 36, and 48 months, respectively. For instance, a patient was 39 years old at the first diagnosis, and this patient was classified as osteosarcoma and has undergone surgery. Red lines and dots are drawn upward to determine the points received by each variable; the sum (170) of these points is located on the total points axis, and a line is drawn downward to the survival axes to determine that the probability of overall survival at 24 months was 43%.

**Table 4:** Univariate and multivariate Cox regression analysis for identification independent prognostic factors in POSNs patients with DM.

**Variables**	**Univariate analysis**			**Multivariate analysis**		
	**HR**	**95%CIs**	***P*–value**	**HR**	**95%CIs**	***P*–value**
Age						
<40 years	Reference			Reference		
40–59 years	1.39	0.85–2.29	0.191	1.68	0.9–3.16	0.1048
≥60 years	3.01	1.88–4.81	<0.001	4.64	2.54–8.49	<0.001
Sex						
Female						
Male	1.02	0.69–1.5	0.93			
Race						
Black						
Other	0.65	0.25–1.7	0.378			
White	0.74	0.39–1.43	0.372			
Grade						
Low (well differentiation)						
High (poor differentiation)	1.63	0.69–3.8	0.262			
Unknown	0.78	0.34–1.81	0.562			
Histological type						
Osteosarcoma						
Chondrosarcomas	0.46	0.26–0.83	0.01	0.33	0.18–0.62	<0.001
Ewing sarcoma	0.26	0.17–0.42	0	0.38	0.22–0.67	<0.001
Chordoma	0.18	0.06–0.5	0.001	0.1	0.03–0.3	<0.001
Other	0.51	0.24–1.07	0.076	0.68	0.31 −1.52	0.3503
T stage						
T1						
T2	0.99	0.65–1.51	0.963			
T3	1.24	0.64–2.4	0.533			
N stage						
N0						
N1	1.04	0.6–1.8	0.881			
Tumor size						
<5 cm						
5–10 cm	0.49	0.24–0.98	0.043	0.55	0.26–1.18	0.1252
>10 cm	0.72	0.37–1.41	0.334	0.8	0.38–1.66	0.5457
Surgery						
No						
Yes	0.58	0.37–0.91	0.016	0.49	0.31– 0.79	0.0035
Radiotherapy						
No						
Yes	0.62	0.42–0.9	0.012	0.82	0.54–1.25	0.3651
Chemotherapy						
No						
Yes	1.08	0.63–1.85	0.768			

**Figure 9:**
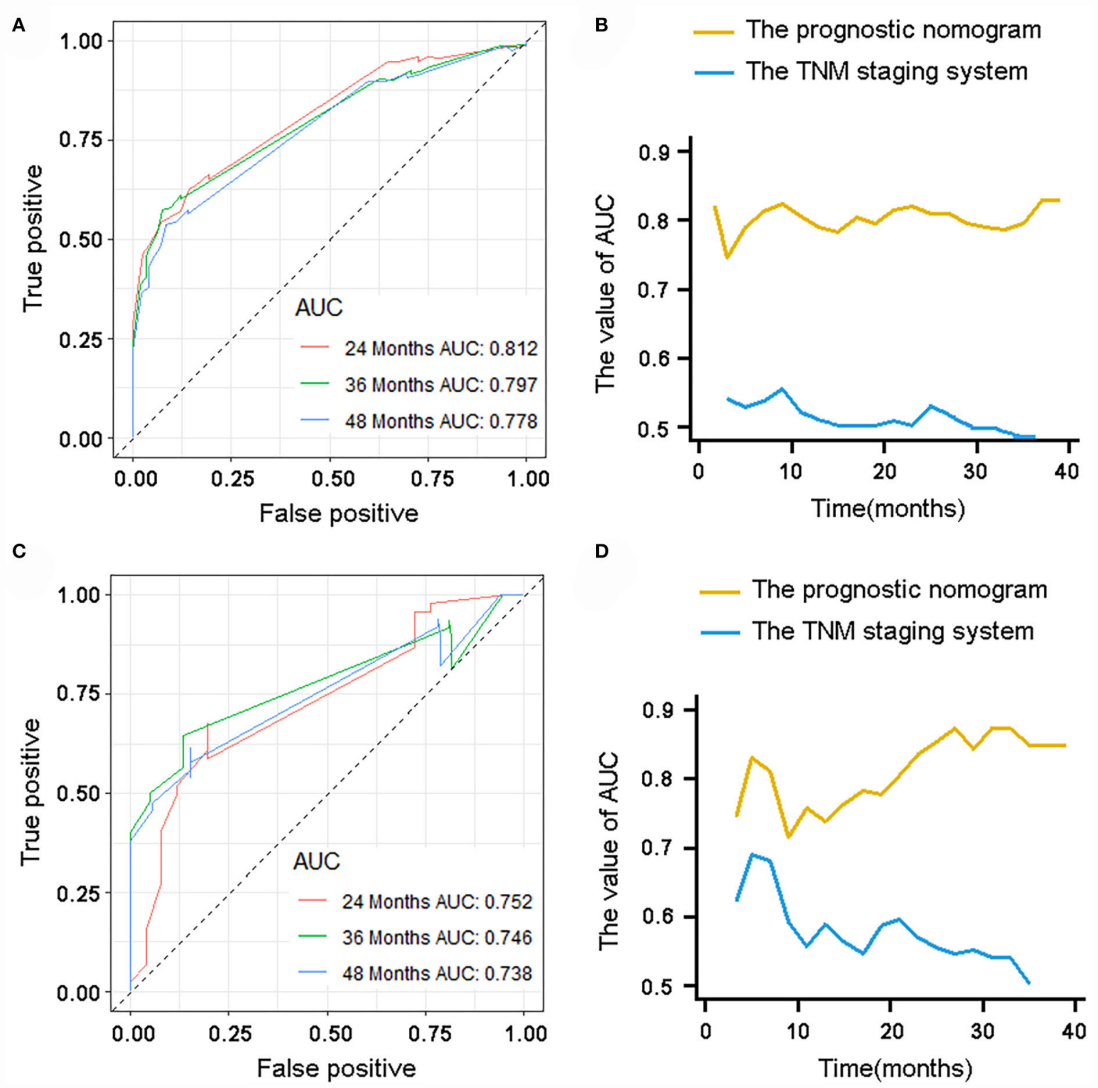
ROC curve analysis of the nomogram for 24, 36, and 48 months in the training set **(A)** and the validation set **(C)**. Time-dependent ROC curves for comparison of the discriminative ability between the nomogram and TNM staging system in the training set **(B)** and validation set **(D)**.

**Figure 10:**
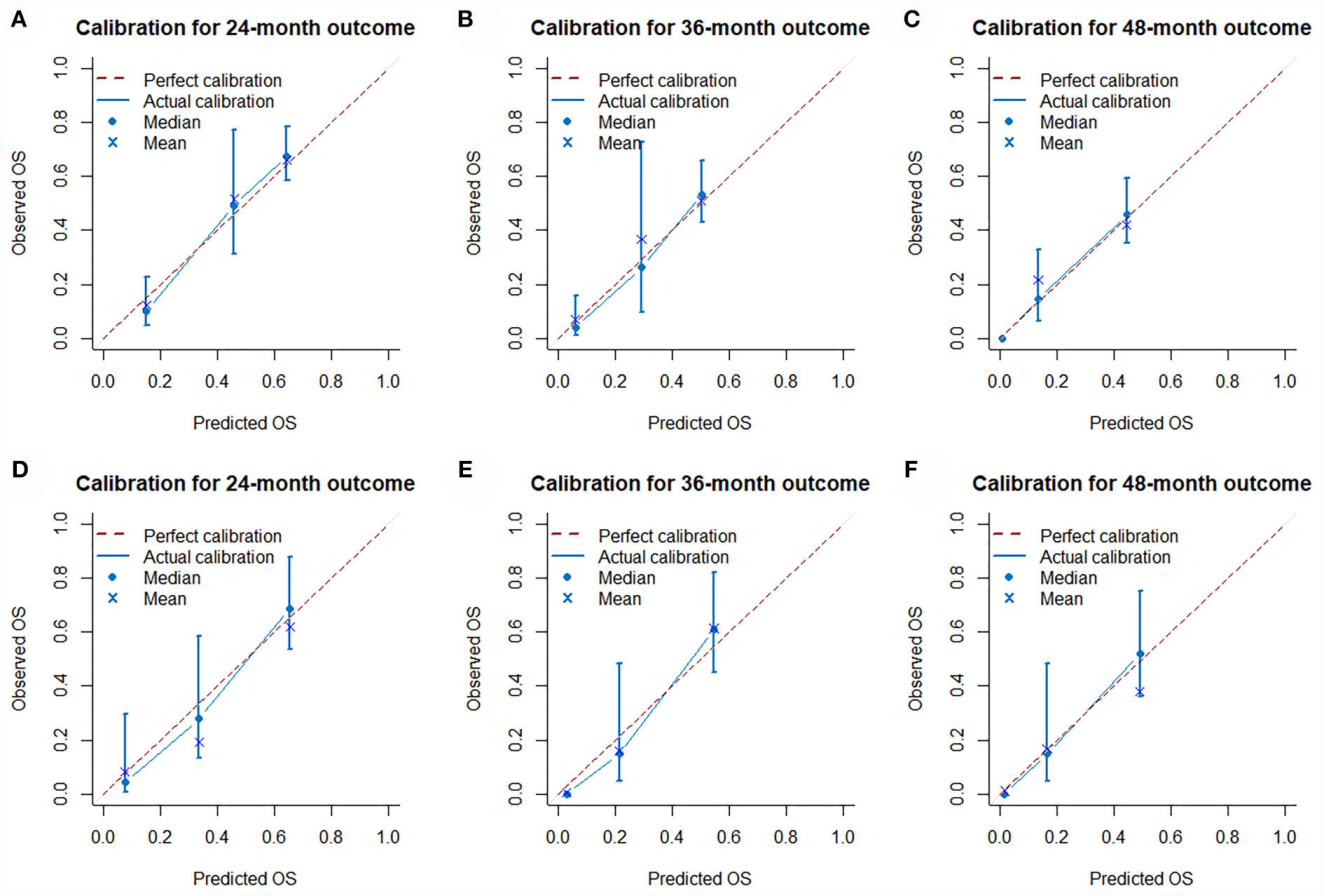
Calibration curves of 24- **(A)**, 36- **(B)**, and 48-month **(C)** OS in the training set and 24- **(D)**, 36- **(E)**, and 48-month **(F)** OS in the validation set.

**Figure 11:**
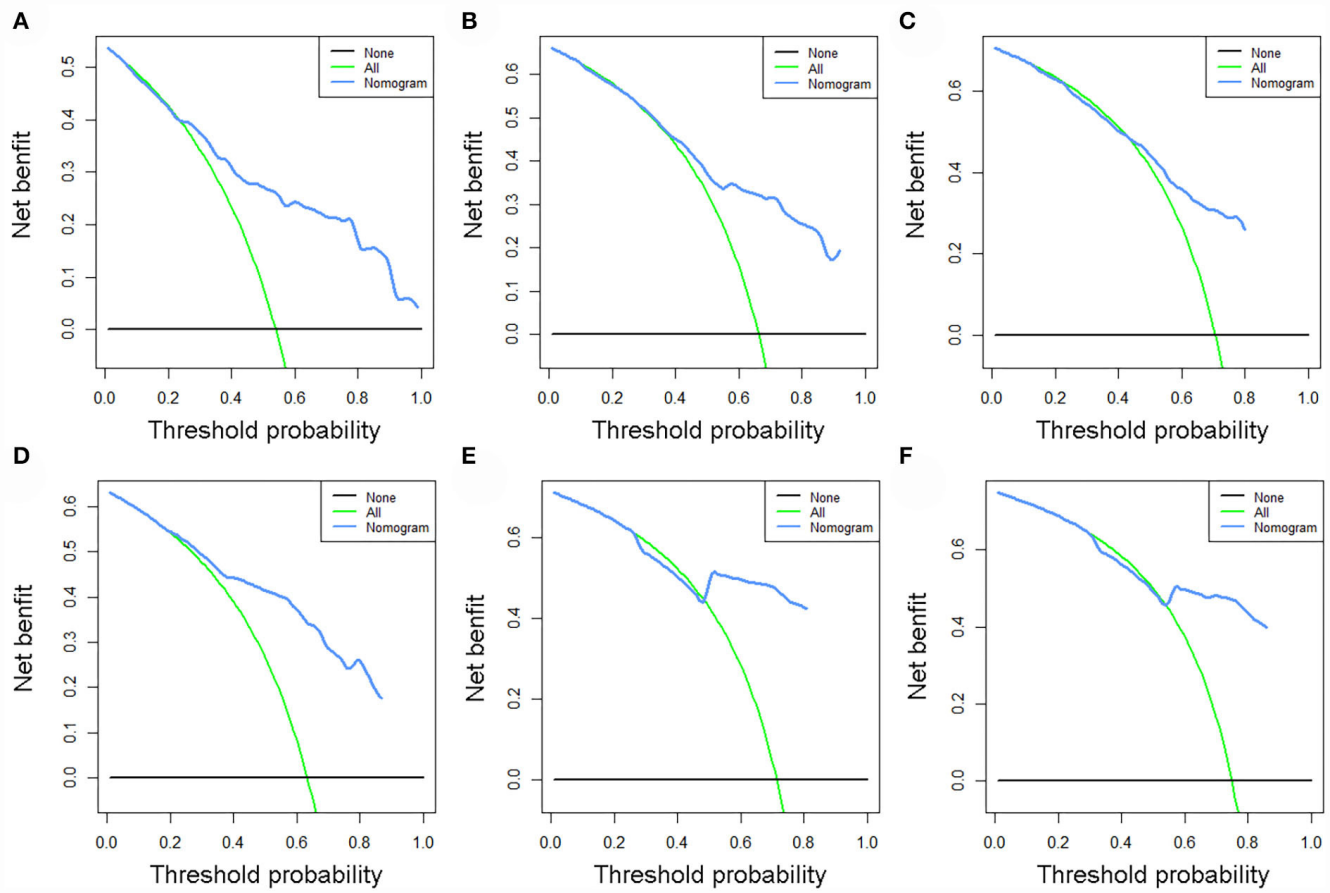
DCA curves of 24- **(A)**, 36- **(B)**, and 48-month **(C)** OS in the training set and 24- **(D)**, 36- **(E)**, and 48-month **(F)** OS in the validation set.

**Figure 12:**
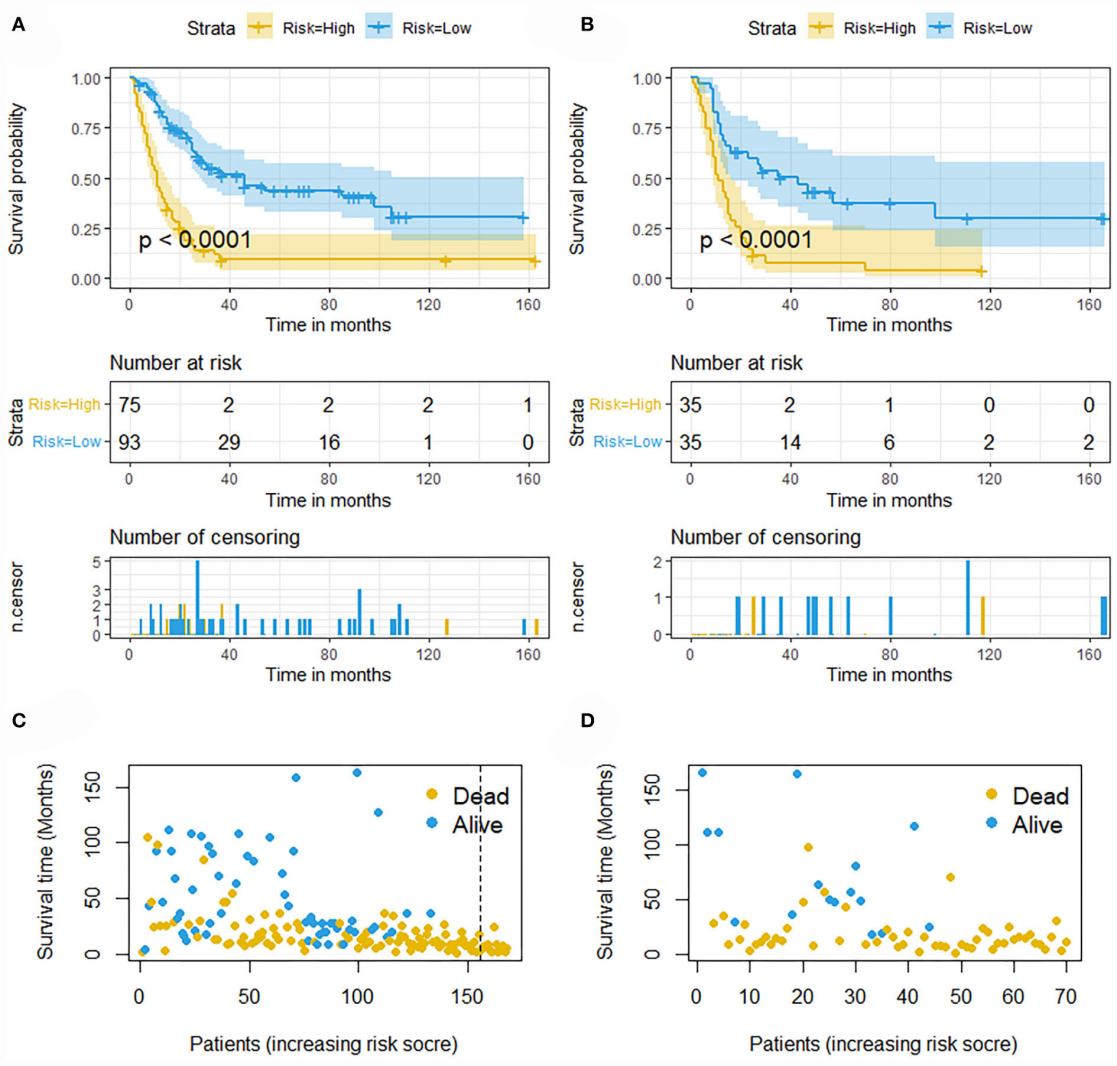
Kaplan–Meier survival curves of two mortality risk subgroups in the training set **(A)** and validation set **(B)**. Kaplan–Meier survival status analysis for patients with POSNs with DM in the training set **(C)** and validation set **(D)**.

The validation of the novel nomogram showed its excellent predictive accuracy. The overall performance of the nomogram was assessed, producing a Harrell's concordance index (C-index) of 0.740 (95% CI: 0.644–0.836) in the training set and 0.710 (95% CI: 0.579–0.841) in the validation set, indicating the adequate discriminative ability of this prediction model. The values of the AUC for OS probability at 24, 36, and 48 months were 0.812, 0.797, and 0.778 (Figure 9A) in the training set and 0.752, 0.746, and 0.738 (Figure 9C) in the validation set. In addition, the time-dependent ROC curves revealed the discriminative ability of the new proposed nomogram was significantly higher than that of the existing TNM staging system (Figures 9B,D). In the analyses of NRI and IDI, the nomogram better performed than the TNM staging system (Table 5). In addition, the calibration plots demonstrated good consistency between the predicted OS probability by nomogram and actual OS in both sets (Figure 10). The DCA curves suggested preferable positive net benefit and strong clinical usefulness of the novel model (Figure 11). Subsequently, we calculated the total point of each patient with POSNs with DM and divided them into low-risk and high-risk groups based on a median score of 145. The K-M survival curves and survival status analysis showed that patients with POSNs with DM who were at high risk had a worse prognosis than those who were at low risk in both training and validation sets (*P* < 0.0001) (Figure 12), suggesting the prognostic nomogram established in our study was expected to be an effective tool in stratifying patients with POSNs with DM according to the estimated risk of mortality.

**Table 5:** NRI and IDI of the prognostic nomogram for POSNs patients with DM compared with TNM staging system.

**Index**	**Training set**			**Validation set**		
	**Estimate**	**95%CI**	***p*–value**	**Estimate**	**95%CI**	***p*–value**
**NRI (vs. AJCC TNM staging)**						
For 24–month OS	0.689	0.335–0.946	<0.001	0.537	0.161–0.912	<0.001
For 36–month OS	0.472	0.120–0.671	<0.001	0.603	0.362–0.843	<0.001
For 48–month OS	0.253	0.059–0.546	<0.001	0.598	0.381–0.812	<0.001
**IDI (vs. AJCC TNM staging)**						
For 24–month OS	0.221	0.174–0.268	<0.001	0.336	0.270–0.402	<0.001
For 36–month OS	0.197	0.155–0.239	<0.001	0.310	0.250–0.370	<0.001
For 48–month OS	0.180	0.141–0.219	<0.001	0.290	0.235–0.345	<0.001

**Figure 13:**
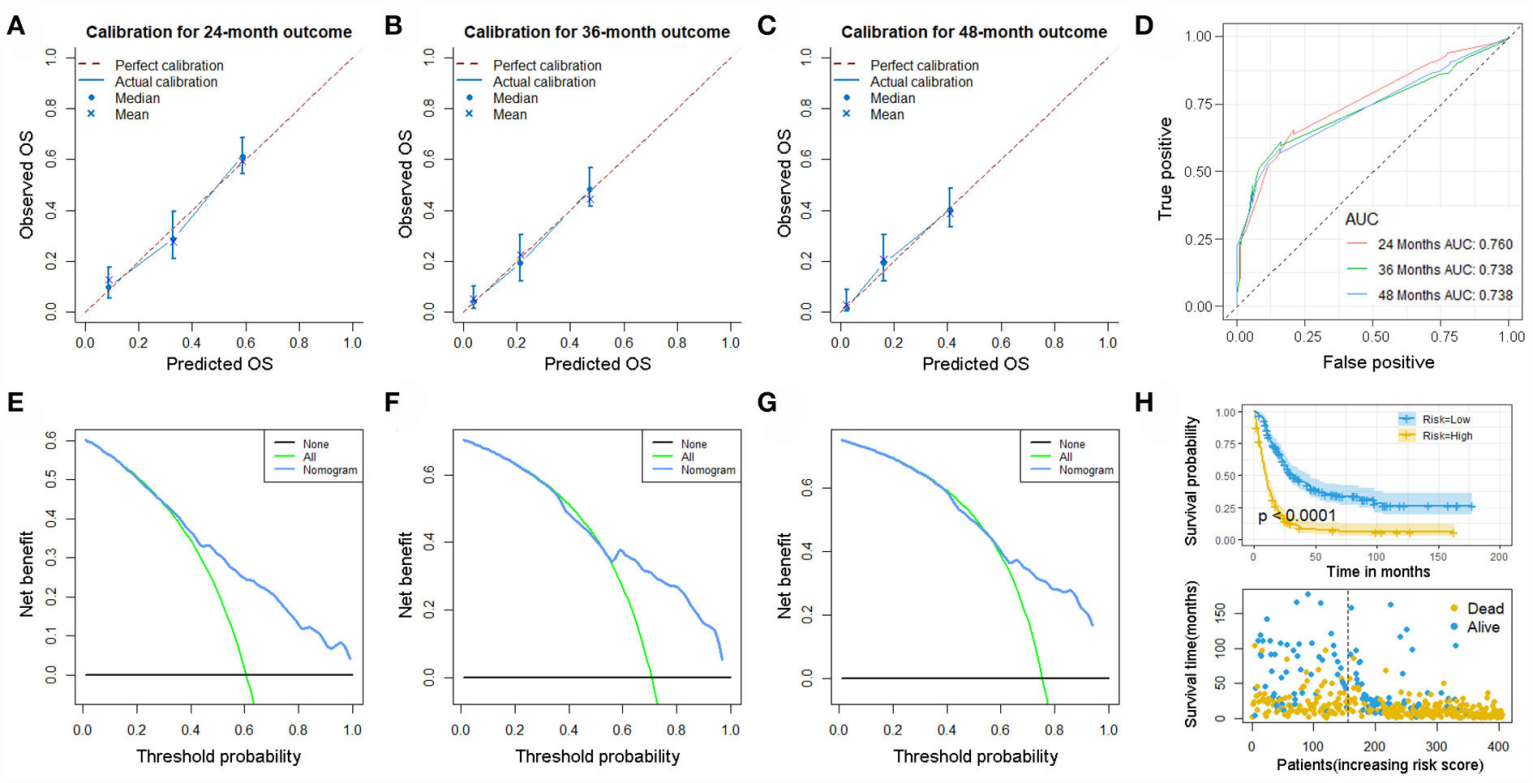
Calibration curves of 24- **(A)**, 36- **(B)**, and 48-month **(C)** OS; ROC curves **(D)**; DCA curves of 24- **(E)**, 36- **(F)**, and 48-month **(G)** OS; and Kaplan–Meier survival analysis **(H)** in the expanded set.

As previous mentioned, we re-screened appropriate patients from the SEER database to form a new set for validation of our prognostic nomogram. Accordingly, 405 patients were included in this expanded validation set, and this nomogram was validated again. The calibration curves suggested good agreement between predicted values and observational values, and the result of DCA indicated this nomogram could be a useful clinical tool in predicting OS of patients with POSNs with DM. In addition, the values of AUC for 24-, 36-, and 48-month OS prediction were 0.760, 0.738, and 0.738 in this set. Moreover, the K-M survival curve suggested that patients in the low-risk group had a higher survival probability than those in the high-risk group (*P* < 0.0001) (Figure 13).

## Discussion

Metastasis is considered one of the most notable prognostic indicators of POSNs, and it is incorporated into almost all previously reported prognostic models established for patients with initially diagnosed POSNs (19, 20). Not only in POSNs but also in other malignancies, as soon as patients develop metastatic disease, their survival rate will drop dramatically compared with their counterparts without DM (24–26). In the present study, 238 (17.70%) cases were confirmed to have distant organ metastasis at the time of initial diagnosis, as shown in Figure 2. The patients with POSNs with DM eventually showed significantly worse prognosis than those without synchronous DM (*P* < 0.05). A few reasons may account for such discrepancies. First, patients with POSNs with synchronous DM seem less receptive to the advice of complete surgical removal due to its special anatomical location and advanced stage of disease, thus delaying the appropriate timing or even missing out on the opportunity of receiving curative surgery, which ultimately results in the reduction of survival in these patients (27, 28). In addition, patients with DM usually suffer from more serious debilitating muscle-wasting syndrome (also known as cachexia), which drastically diminishes tolerance to antitumor treatment, which may accelerate death in the absence of effective therapeutic interventions (29). Furthermore, the presence of DM is generally accompanied by chemoresistance, resulting in a decline in treatment efficacy at the same dose of antineoplastic agents (30, 31). Owing to the significant stresses provided by the different internal environment, including antitumor immunity, inflammatory responses, and the formation of reactive oxygen species, those tumor cells that metastasize to other tissues and achieve successful colonization would therefore acquire stronger vitality and higher potential resistance (32, 33). Although the survival outcome is extremely poor in patients with POSNs with DM, the early detection of DM is crucial for patients with POSNs to improve prognosis. Therefore, it is of considerable interest to identify which patients with POSNs are at high risk of DM and to accurately predict the survival rate of patients with POSNs with DM, which may help clinicians determine individualized treatment strategies in this special group.

Although Zhou et al. had conducted one of the largest retrospective studies aimed at constructing a reliable risk assessment system for patients with initially diagnosed osseous spinal and pelvic tumors, it was noteworthy that this was not specifically designed for patients with metastatic disease (34). In our study, we developed two nomograms for the prediction of the risk and prognosis in patients with POSNs with DM. Overall, the predictive efficiency and practical value of these novel models were satisfactory, and the results of the time-dependent ROC curve, NRI, and IDI showed that the newly proposed prognostic nomogram had better discriminative ability than the conventional AJCC TNM staging system. Furthermore, dynamic probability calculators based on the two nomograms were further developed to improve the convenience of the practical application of these two nomograms. With the help of this online version of the nomogram, clinicians can dynamically predict the probability of OS for patients with POSNs with DM at various time points.

Patients with poorly differentiated POSNs were more prone to suffer from metastatic disease; a reasonable explanation was that poorer differentiation meant greater local aggressiveness, which might accelerate tumor progression and eventual metastasis to distant organs (35). Moreover, the histological type of primary tumor was found to be significantly correlated with synchronous DM. As a highly aggressive bone tumor, the rate of DM for Ewing sarcoma at initial diagnosis was reported to be around 15-20% (36), while chordoma was considered as a low-grade, slow-growing malignancy that had a limited tendency to metastasize, with the rate of DM of spinal chordoma of approximately 3–7% (37). In the present study, we observed that patients with spinal Ewing sarcoma showed the highest potential for metastasis, whereas patients with spinal chordoma were least likely to develop DM, which was consistent with the previously reported tendency of DM in different types of bone tumors. In addition, this study also pointed out that T stage and N stage were important factors influencing the development of DM. This was not surprising since the higher T stage frequently implied more vascular involvement and longer tumor growth time before diagnosis. Meanwhile, the tumor cells would divide further out of control as time advanced (38). All of these might promote the occurrence of metastatic disease. Although regional lymph node metastasis (LNM) was reported to be associated with poor prognosis, it was rarely observed in primary malignant bone tumor, with an incidence ranging from 3 to 10% (39, 40). Our analysis indicated involvement of the regional node played a fundamental role for DM in patients with POSNs. Therefore, orthopedic surgeons were advised to pay more attention to suspicious tumors and to be more aggressive in performing regional lymph node biopsies in clinical practice.

Moreover, age, histological type, and surgery were determined to be independently associated with the OS rate of patients with POSNs with synchronous DM. Age at diagnosis was widely reported to be an important prognostic factor in primary malignant bone tumors (41, 42). This study suggested that prognosis of patients with metastatic POSNs deteriorated gradually with age. This might be because older patients were often accompanied by reduction in physiological reserve and some underlying diseases, such as diabetes, arteriosclerosis, and hypertension, which might aggravate postoperative complications (43, 44). Moreover, frailty caused by advanced age often made these patients less tolerant to antitumor therapy, which would result in a significant reduction in survival rates (29). In addition, the histological type was found to be the predictor that had the most significant impact on survival among all types of POSNs, and patients with osteosarcoma experienced the worst prognosis, while patients with chordoma had the most satisfactory survival outcome (HR = 0.1, 95% CI:0.03–0.3, *P* < 0.05). This finding was similar to the result of a previous study focusing on the entire population of patients with POSNs (34). To the best of our knowledge, the current orthopedic study suggested that the cornerstone of treatment for primary malignant bone tumors was surgical removal of the tumor, including wide excision or amputation (45). However, surgery for patients with POSNs posed a unique set of challenges due to the specificity of the anatomical position of tumor and the necessity of adjacent anatomical structures (46, 47). Nonetheless, our study still demonstrated that patients with metastatic POSNs who received primary tumor resection had a better survival rate than those who did not. This was in line with a previous study that showed that surgery significantly improved survival in spinal chondrosarcoma patients with DM (48). Although primary tumor resection provided significant survival benefit in patients with metastatic POSNs, a recent study of Kazim SF et al. suggested that the frailty status was more important than age in predicting postoperative outcomes for patients with POSNs, which helped clinicians more accurately determine whether a patient would do well in surgery of spinal tumor (49). As patients with POSNs with DM tended to have more severe cachexia and frailty, we recommended that surgeons should carefully assess the overall condition of these patients to determine the risks and benefits of proposed surgical intervention. All in all, two visualized nomograms incorporating aforementioned predictors promised to be useful tools in quantifying the risk of DM in patients with POSNs and predicting the survival rate of patients with POSNs with DM, eventually providing guidance for further personalized clinical management.

This study, similar to other SEER-based studies, still has several limitations, which should be acknowledged. Above all, it is difficult to avoid selective bias because this study is a retrospective one. In addition, there is a lack of external data from different regions due to the rarity of POSNs; therefore, further validation through prospective studies is needed to verify whether these results are generally applicable. Third, because of the limited parameters recorded in the SEER database, we are unable to consider other clinical factors and biomarkers not collected in the database, which may have affected the outcomes, such as target therapy, postoperative complications, gene expression, and chromosomal alteration.

## Conclusion

In our study, two easy-to-use nomograms and their online versions were established to identify patients with POSNs at high risk of DM and then estimate survival outcome of these patients, which might help orthopedic surgeons better develop clinical management and treatment strategies.

## Data availability statement

The raw data supporting the conclusions of this article will be made available by the authors, without undue reservation.

## Author contributions

YT and DZ conceived and designed the study and revised the manuscript. YT and ZH collected the clinical data and literature review. YT and LJ conducted the statistical analysis. YT, YP, and YG generated figures and tables. YT wrote the manuscript. DZ supervised the research. All authors critically read the manuscript to improve the intellectual content and read and approved the final manuscript.

## Funding

This work was supported by the Jilin Science and Technology Program, China (20190201282JC).

## Conflict of interest

The authors declare that the research was conducted in the absence of any commercial or financial relationships that could be construed as a potential conflict of interest.

## Publisher's note

All claims expressed in this article are solely those of the authors and do not necessarily represent those of their affiliated organizations, or those of the publisher, the editors and the reviewers. Any product that may be evaluated in this article, or claim that may be made by its manufacturer, is not guaranteed or endorsed by the publisher.

## Abbreviations

POSNs: primary osseous spinal neoplasms
DM: distant metastasis
CT: computed tomography
MDP: methylene diphosphonate
PET/CT: positron emission computed tomography
ICD-O-3, International Classification of Diseases for Oncology: third edition
K-M: Kaplan–Meier
OS: overall survival
CSS: cancer-specific survival
SEER, Surveillance, Epidemiology: and End Results
AJCC: American Joint Committee on Cancer
CI: confidence interval
HR: hazard ratio
OR: odds ratio
ROC: receiver operating characteristic
AUC: area under curve
DCA: decision curve analysis
NRI: net reclassification improvement
IDI: integrated discrimination improvement.
